# A Comparative Study of Gallium-, Xenon-, and Helium-Focused Ion Beams for the Milling of GaN

**DOI:** 10.3390/nano13212898

**Published:** 2023-11-03

**Authors:** Shuai Jiang, Volkan Ortalan

**Affiliations:** Department of Materials Science and Engineering, University of Connecticut, Storrs, CT 06269, USA; shuai.2.jiang@uconn.edu

**Keywords:** gallium nitride, focused ion beam, milling profiles, transmission electron microscopy, molecular dynamics

## Abstract

The milling profiles of single-crystal gallium nitride (GaN) when subjected to focused ion beams (FIBs) using gallium (Ga), xenon (Xe), and helium (He) ion sources were investigated. An experimental analysis via annular dark-field scanning transmission electron microscopy (ADF-STEM) and high-resolution transmission electron microscopy (HRTEM) revealed that Ga-FIB milling yields trenches with higher aspect ratios compared to Xe-FIB milling for the selected ion beam parameters (30 kV, 42 pA), while He-FIB induces local lattice disorder. Molecular dynamics (MD) simulations were employed to investigate the milling process, confirming that probe size critically influences trench aspect ratios. Interestingly, the MD simulations also showed that Xe-FIB generates higher aspect ratios than Ga-FIB with the same probe size, indicating that Xe-FIB could also be an effective option for nanoscale patterning. Atomic defects such as vacancies and interstitials in GaN from He-FIB milling were suggested by the MD simulations, supporting the lattice disorder observed via HRTEM. This combined experimental and simulation approach has enhanced our understanding of FIB milling dynamics and will benefit the fabrication of nanostructures via the FIB technique.

## 1. Introduction

The focused ion beam (FIB) is a powerful technique widely used in materials science and the electronics industry for purposes such as TEM sample preparation, fabrication of micro and nanoscale patterns, and circuit editing [1,2,3,4,5,6]. The traditional gallium FIB (Ga-FIB) uses a gallium-based liquid metal ion source to deliver a gallium ion beam to remove materials, while the emerging plasma-FIB (Xe-FIB) employs heavier ion species like xenon (Xe) as the ion source, which is ideal for the removal of large volumes due to faster milling rates and helps eliminate Ga ion-induced contaminations during the Ga-FIB milling process [7,8]. In addition to these two FIB systems, the latest helium-focused ion beam (He-FIB) system, which utilizes a gas field ionization source, has been showing great potential in the fabrication of nanoscale patterns due to its atomically sharp and extremely bright ion source [9,10]. FIB milling has been widely applied to metals and various alloys; however, the demand for FIB processing of semiconductor materials like silicon (Si), silicon carbide (SiC), and gallium nitride (GaN) has been increasing [11,12,13,14,15,16]. GaN is a wide-bandgap semiconductor and a good candidate for high-power applications and light-emitting diodes [17,18,19]. For enhanced performance in GaN-based devices, the development of improved milling techniques is essential. The FIB is an excellent processing technique for GaN since it is hard to obtain smooth mirror facets with conventional processing methods [20,21]. Chyr et al. conducted a series of experiments to study the milling rates and sputtering yields of GaN for the Ga ion beam and the Au-ion beam under various experimental conditions [13,22]. Lin et al. focused on sputtered profiles of GaN substrates with Ga-FIB from experimental and simulation perspectives [23]. In addition to experiments, various simulation methods have been applied to study the sputtering processes of different substrates by ion beams. Stopping and Range of Ions in Matter (SRIM) is a software package based on the Monte Carlo method to calculate the transport of ions in matter [24]. However, the Monte Carlo method assumes the target to be amorphous; therefore, it is not suitable for orientation-dependent studies of single crystals. Molecular dynamics (MD) simulations, on the other hand, can overcome this limitation [25,26]. MD simulations are widely used for the bombardment of solids by energetic particles because of their capability to observe the interactions between atoms at the atomic level [27]. A series of MD simulations have been applied to study the interaction between focused ion beams and substrates. Russo et al. simulated the bombardment of a silicon surface with gallium ion beams of 2 kV and 30 kV to explain the mechanism of the V-shaped trench formation [28]. Satake et al. compared the differences among various potential functions in simulating the sputtering process [29]. Using MD simulations, Tong et al. discussed the relationship between the thickness of the amorphous layer formed on the surface of the diamond during the FIB treatment and the milling voltage [30].

In this study, three FIB systems with different ion sources, Ga-FIB, Xe-FIB, and He-FIB, were utilized to study the milling of single-crystal GaN. The utilization of these three sources was motivated by their prominence as mainstream FIB systems and their relative accessibility within the research community. The milling voltage and current were kept close to each other during the experiments. Cross-sectional samples were then prepared using a Ga-FIB system for the analysis of formed trenches via transmission electron microscopy (TEM). The final profiles created by the ion beams were characterized using annular dark-field scanning transmission microscopy (ADF-STEM) and high-resolution transmission electron microscopy (HRTEM). Furthermore, MD simulations were performed to model the sputtering processes of GaN with high-energy Ga, Xe, and He particles at the atomic scale.

## 2. Materials and Methods

**Figure 1:**
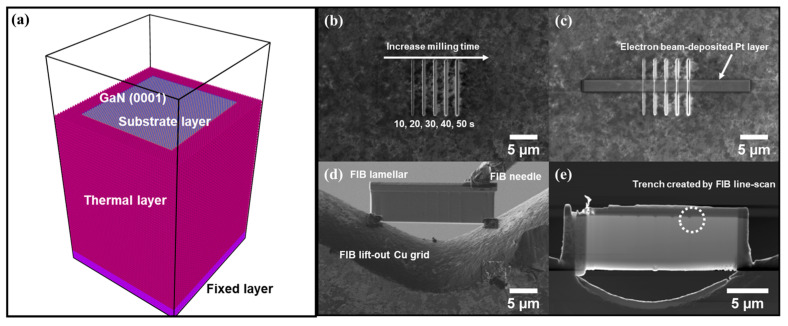
The perspective view of the MD simulation model, the FIB milling process, and preparation of TEM-FIB lamellar. (**a**) The model is composed of a substrate layer, a thermal layer, and a fixed layer. The GaN (0001) facet is normal to the direction of the bombardment of the ion particles. (**b**) Trenches milled via the FIB line-scan mode (30 kV, 42 pA) at different milling times (10 s, 20 s, 30 s, 40 s, 50 s). (**c**) Pt protection layer with electron beam deposition. (**d**) FIB lamellar attached to the lift-out Cu grid. (**e**) Thinned FIB lamellar showing the cross-sectional profiles of trenches created by FIB line scan.

In these experiments, Helios PFIB Dual Beam, Helios 460F1 Dual Beam, and Orion Nanofab instruments were employed for the milling of the GaN (0001) facet with Xe, Ga, and He ions, respectively. The FIB line-scan mode was used to obtain ion-induced surface features, and the lamellae for TEM analysis were prepared using the Ga-FIB system, as illustrated in Figure 1. The length of the line was 10 µm, and the milling time was selected between 10 s and 50 s with increments of 10 s. The applied voltage was kept constant at 30 kV during the line scan, and currents for Ga ions, Xe ions, and He ions were 42 pA, 42 pA, and 22 pA, respectively, which were measured with the calibrated Faraday cup inside the FIB systems. The current in the He-FIB was set to 22 pA since it is the maximum current available in the instrument. The probe sizes of the Ga- and Xe-focused ion beams in our systems were measured using the “rise distance” method [31]. To determine the probe sizes of the Ga-FIB and Xe-FIB, the rise distances of secondary electron signals were measured when the beam was scanning along the cleaved edge of a single-crystal silicon wafer tilted at 7º with respect to the directly focused ion beam. The probe size is defined as the distance from 25% to 75% of the difference between maximum and minimum values. The probe sizes for the Ga-FIB and Xe-FIB under given conditions (30 kV, 42 pA) were measured to be 21 nm and 62 nm, respectively, as shown in Figure S1. For the preparation of the FIB lamellae used in TEM analysis, a 100 nm thick platinum (Pt) or carbon (C) protection layer was first deposited onto the GaN surface through electron beam deposition to protect the surface features, and then a 2 µm Pt layer was deposited via gallium ion beam-assisted deposition (Figure 1c). The cross-sectional FIB lamellae were prepared using the widely used lift-out technique afterward [32]. The lamellae were then analyzed with Talos F200X S/TEM (Thermo Fisher Scientific Inc., Waltham, MA, USA).

Molecular dynamics simulations were performed by the Large-scale Atomic/Molecular Massively Parallel Simulator (LAMMPS) program using Ziegler–Biersack–Littmark (ZBL) and Tersoff potentials [33,34]. The ZBL potential was used to describe the interactions between the ion particles and GaN, such as Xe-Xe, Ga-Ga, He-He, Xe/Ga/He-Ga, and Xe/Ga/He-N interactions. The Tersoff potential was used to describe the forces within GaN, like Ga-Ga, Ga-N, and N-N pairs. The size of the single-crystal GaN with (0001) surface normal to the direction of the ion beam bombardment is 14.4 nm × 14.9 nm × 18.7 nm. As shown in Figure 1a, the system was composed of three layers: a fixed layer, a thermal layer, and a substrate layer. The substrate layer interacts with the incoming Xe, Ga, and He particles from 1 nm above the top surface, while the thermal layer holds the temperature of the system at 300 K. The fixed layer is static during sputtering. First, the structure was relaxed at 300 K for 60,000 iterations through the NVT thermostat. Then, 500 Gaussian-distributed ion particles were released one by one, interacting with the (0001) surface of the GaN substrate. The particle energies were set to 2 keV instead of 30 keV used in the experiments to reduce the computational cost, and the velocities of Ga, Xe, and He particles were set to 744 A/ps, 542 A/ps, and 3104 A/ps, respectively, to match the 2 keV energy. An adaptive timestep of 0.002–1 fs was used to ensure that no atom would move further than 0.02 Å per iteration, and the timestep between two adjacent ion particles was set to 60,000 to make sure that the substrate layer had been sufficiently damped. The substrate is cooled back to 300 K before the next bombardment event. A single bombardment event takes about 60 ps.

## 3. Results

### 3.1. Damage Profiles by FIBs with Different Ions

**Figure 2:**
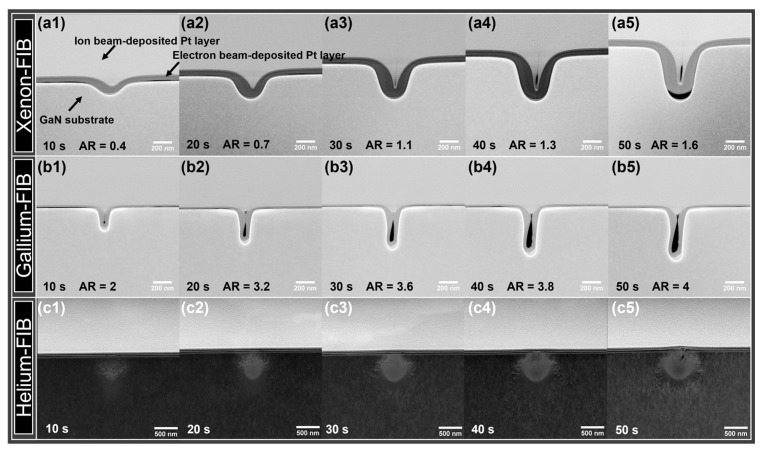
ADF-STEM images showing the trench profiles created by (**a1**–**a5**) Xe-FIB (30 kV, 42 pA), (**b1**–**b5**) Ga-FIB (30 kV, 42 pA), and (**c1**–**c5**) He-FIB (30 kV, 22 pA) line-scans at various milling times. AR represents the aspect ratio of the trench and is defined as the ratio between the depth and the width at the half depth of the trench.

The damage profiles of GaN milled with the xenon, gallium, and helium beams were characterized by Annular Dark-Field STEM (ADF-STEM) and Energy-Dispersive X-ray Spectroscopy (EDS) mapping to see the evolutions of the trenches with different milling times, as shown in Figure 2 and Figure S2. Under the experimental condition (30 kV, 42 pA), higher aspect ratios (AR) were obtained for trenches created by the gallium-focused ion beam (AR: 2 (10 s)–4 (50 s)) compared to that produced by the xenon-focused ion beam (AR: 0.4 (10 s)–1.6 (50 s)). As can be seen from the images, the difference is mainly because the xenon-focused ion beam always yields a larger opening width, which eventually leads to a relatively low aspect ratio. We also noticed that the ratio of the opening width of Xe-trench to that of Ga-trench is about 3 from 20 s to 50 s, which is close to the ratio of the probe size of the xenon (62 nm) focused ion beam to that of the gallium (21 nm) focused ion beam at 30 kV, 42 pA (Figure S1). For the helium-focused ion beam, Z-contrast High-Angle ADF-STEM imaging was applied to obtain images with better contrast. As can be seen in Figure 2(c1–c4), the milling did not occur till 40 s at 30 kV and 22 pA. A moment of imminent rupture of the top surface was captured at 50 s of milling (Figure 2(c5)), suggesting a low milling efficiency of He-FIB under these conditions. Under all experimental conditions, a semicircle-like region with brighter contrast was found, and this region expanded with increasing milling time. High-resolution TEM (HRTEM) characterizations, together with molecular dynamics (MD) simulations, suggest that this region is rich in vacancies and interstitials and will be discussed in detail later.

**Figure 3:**
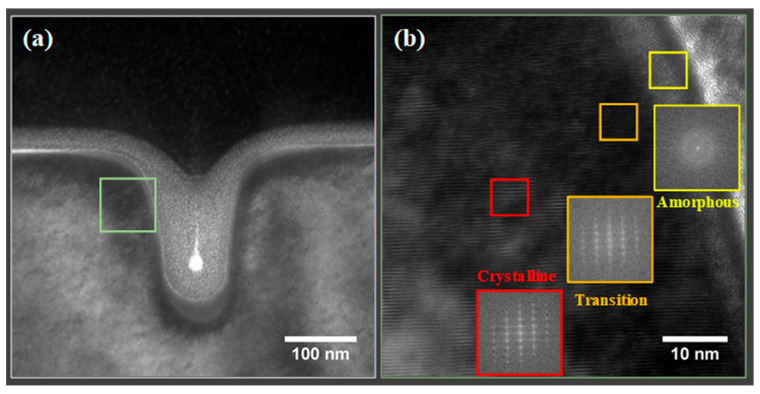
TEM images (**a**,**b**) of milling profile by Ga-FIB (30 kV, 42 pA, 10 μm line scan for 10 s) and the corresponding FFTs of different regions in (**b**) showing three regions: Crystalline, Crystalline-Amorphous Transition, Amorphous.

To gain a better understanding of the milling process from the nanoscopic scale, HRTEM images were collected to see the lattice changes at the milling surface. Figure 3 shows the TEM images of the sample after the Ga ion beam milling, as well as the corresponding Fast Fourier Transforms (FFTs) computed from various regions in the HRTEM image. As can be seen in Figure 3b, there are three regions with different contrasts in the Ga-distributed area. The FFTs (insets of Figure 3b) of different areas in the HRTEM images verified the state of different regions: a crystalline region with sharp FFT peaks, a transition region with broadened FFT peaks, and an amorphous region with no sharp peaks. Similarly, three regions with crystalline-to-amorphous transition were also found in the Xe-FIB-milled GaN (Figure S3). The distribution of the amorphous regions (Figure 3a) also suggests that the amorphous region not only exists at both sides and the bottom of the trench but extends towards the top surface of the crystal. The amorphization of the top surface is mainly due to the redeposition of sputtered materials, which was also replicated by the MD simulations.

**Figure 4:**
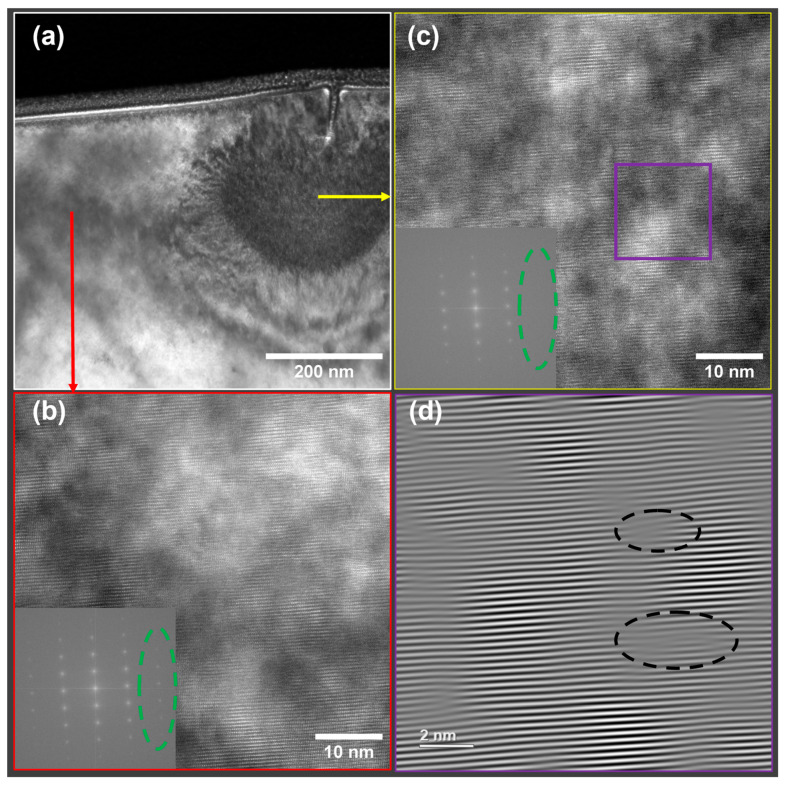
TEM image (**a**) of milling profile by He-FIB (30 kV, 22 pA, 10 μm line scan for 50 s) and HRTEM images (insets are the corresponding FFTs) showing different regions: (**b**) Crystalline, (**c**) Defected, (**d**) Fourier-filtered (0002) diffraction high-magnification image of a cropped region in (**c**).

To identify the nature of the semicircle-like region in the He-FIB-milled GaN, HRTEM images from both the semicircle-like and unaffected regions were obtained. Figure 4 shows a bright-field TEM image of the sample after 50 s of He ion milling and HRTEM images collected from different regions. The crystallinity of the unaffected crystalline region was confirmed by the HRTEM images with clear lattice contrast and sharp FFT peaks (Figure 4b). However, in the semicircle-like region (Figure 4c), the lattice contrast was less pronounced, and some FFT spots representing lattice planes with smaller interatomic spacings were missing (marked by the green oval dashed circle), indicating that the lattice disorder might be created by the helium ion beam. Stacking faults and dislocation loops marked by the dark dashed oval circles in Figure 4d further suggested the existence of interstitial defects. The macroscopic crystal expansion around the rupture region in Figure 4a could be ascribed to such interstitial-induced stress [35].

### 3.2. MD Simulations

**Figure 5:**
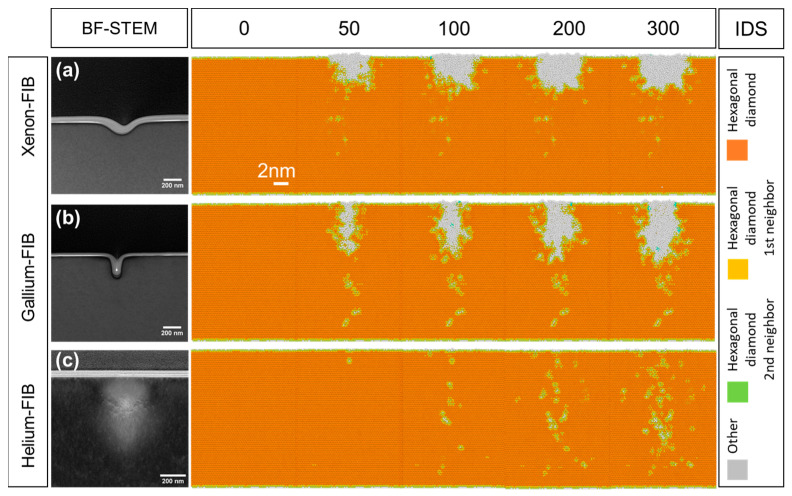
STEM images showing the milling profiles milled for 10 s by (**a**) Xe-FIB with 3 nm, (**b**) Ga-FIB with 1 nm, (**c**) He-FIB with 0.5 nm probe sizes, and the corresponding cross-sectional IDS defect distributions under 0, 50, 100, 200, 300 impacts obtained by MD simulations.

MD simulations were performed to investigate the effect of the ion beam on single-crystal GaN during the FIB process. The probe sizes of the incident particles (Xe and Ga) during simulations were set to be 3 nm and 1 nm to match the ratio of the probe sizes for the Xe-FIB (62 nm) and Ga-FIB (21 nm) during the experiments. The simulations for the He-FIB were performed using typical probe sizes of 0.5 and 1 nm for optimal conditions in the helium ion microscope. The Identify Diamond Structure (IDS) analysis was applied since it can be used to classify the local disorders in cubic and hexagonal diamond structures [36]. Figure 5a–c demonstrates the evolution of the IDS defects under bombardments with different numbers of the incident Xe (3 nm probe), Ga (1 nm probe), and He (0.5 nm probe) particles, respectively. The distribution of the IDS defects is in accordance with the experimental results, elucidating the observed differences in the aspect ratios of the trenches. In addition, in the simulation of the He particles bombarding the GaN surface, a small IDS defect appeared at 1.5 nm from the upper surface of the sample after the bombardment of 50 particles. As the number of particles increases, more IDS defects occur progressively, albeit still very localized, indicating that the milling is considerably less efficient with He ion bombardment compared to that with the Ga and Xe ions, which is in line with the experimental observations.

**Figure 6:**
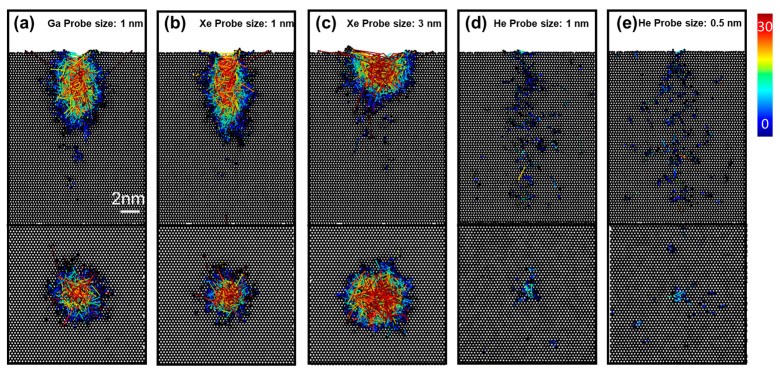
Cross-sectional views of 1 nm thick central slice and top views of 1 nm thick slice at 3 nm below the top surface (for Ga and Xe ions) and 1 nm thick slice from the top surface (for He ion) of GaN structures after 500 impacts of (**a**) Ga with 1 nm, (**b**) Xe with 1 nm, (**c**) Xe with 3 nm, (**d**) He with 1 nm, and (**e**) He with 0.5 nm probe sizes. The arrows with the color gradient represent the magnitude of the displacement vectors of atoms from their static positions, ranging from 0 Å to 30 Å.

To examine whether the experimentally observed variations in the aspect ratios of the trenches are mainly due to the different probe sizes of each focused ion beam, bombardments by Ga, Xe, and He ion beams with various probe sizes were simulated. Figure 6 displays cross-sectional views of a 1 nm thick central slice and top views of a 1 nm thick slice at 3 nm below the top surface of GaN for the Ga and Xe ion beams (Figure 6a–c), and 1 nm thick slice from the top surface of GaN for the He ion beam (Figure 6d,e), after 500 impacts, in addition to the atomic displacement vectors within the substrate. First, bombardments by the Ga and Xe with the same probe size (1 nm) were simulated to explore the differences between the gallium and xenon-focused ion beams in milling GaN substrate (Figure 6a,b). Figure 6b,c shows that for the xenon-focused ion beam, a larger probe size leads to a trench with a larger opening width and a lower aspect ratio. For Xe with a 1 nm probe size, the dimensions (opening width and depth) of the trench milled on GaN (Figure 6b) are close to that of the trench milled with Ga with a 1 nm probe size (Figure 6a), in comparison to the dimensions of the profile milled by Xe with a 3 nm probe size (Figure 6c). Such differences indicate that the probe size is a critical parameter for the aspect ratios of the trenches rather than the ion type for the gallium and xenon-focused ion beams. In addition to the cross-sectional views, the top views of a 1 nm thick slice at 3 nm below the top surface were presented as well. As can be seen from the top views, the atoms of GaN bombarded by Ga tend to move more along the lateral direction compared to that bombarded by Xe when the probe size is set to be 1 nm. This observation implies that Xe-FIB might lead to a higher aspect ratio if the probe sizes of the Xe-FIB and Ga-FIB are the same. Lastly, compared to Xe and Ga, the He ion bombardment did not cause a displacement of the GaN atoms to the extent for which a large trench can be observed (Figure 6d,e). Only a small opening on the upper surface was formed (Figure 6d), which coincides with the rupture of the top region captured in the experiments (Figure 2(c5)). As can be seen from the top views shown in Figure 6d,e, He ions with a 1 nm probe size led to a larger local concentrated region with displaced atoms at the surface compared to that with a 0.5 nm probe size, resulting in a noticeable opening at the surface. In addition to the milling, the redeposition of atoms can be identified by the additional atoms at the top surface near the trench and the displacement vectors (Figure 6a–c), in agreement with the experimental observations (Figures 3a and S3a).

**Figure 7:**
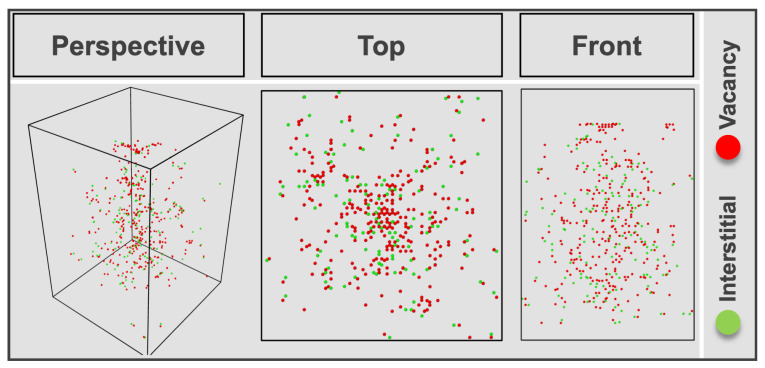
Different views of point defects distribution within the GaN structure after 300 impacts of He with 1 nm probe size obtained by MD simulations.

The Wigner-Seitz defect analysis was applied to identify the distribution of point defects, such as vacancies and interstitials, in the structure via the “OVITO” software (Version 3.9.2) [37] by comparing the structure configuration of GaN after 300 He ion impacts with the initial structure configuration (Figure 7). The distribution of vacancy and interstitial sites throughout the entire structure, extending beyond the local surrounding area of the probe, supports the experimental observations in Figure 4a.

After combining all the experimental data and simulation results, significant differences were found between the Xe-FIB, Ga-FIB, and He-FIB regarding milling single-crystal GaN. Both Xe-FIB and Ga-FIB showed very high efficiency in material removal, while the milling ability of He-FIB was relatively weak. Under the same low current conditions (42 pA), the Ga-FIB can fabricate nanoscale trenches with higher aspect ratios due to its relatively small probe size compared to the Xe-FIB, but the Ga-FIB is inevitably affected by the gallium implantation [38]. As for the Xe-FIB, although it is primarily designed for high-volume milling tasks due to its higher efficiency at higher current levels (nanoampere), our MD results suggest that the Xe-FIB may outperform the Ga-FIB for nanoscale patterning using equivalent spot sizes at low currents (sub-100 picoampere). Given that the Xe-FIB milling avoids gallium implantation, exploring the field of nanoscale patterning with the Xe source deserves further investigation. During the investigation process, HRTEM imaging was found to be a very effective tool for characterizing the local defects created with the ion beam milling, especially for characterizing the oxidation layer that generally existed during the FIB fabrication process. Combined with the FFTs analysis, the crystalline-to-amorphous transition during the FIB milling process can be mapped out as well. It is also important to emphasize that our HRTEM results indicate that the He-FIB can be used as a favorable tool for defect engineering. MD simulations further show the generation of point defects, such as vacancies and interstitials. This observation is consistent with the previous reports on related applications [35]. By controlling the dosage of the helium ions on the sample surface, desired atomic defects can be created, for example, by limiting the defect level to be at the atomic scale [39]. This strategy has been successfully applied in developing high-performance functional materials and devices, such as the ferroelectricity enhancement in HfO_2_ thin films due to oxygen vacancies created with the helium ion bombardment [40] or creation and guiding of stable skyrmions [41], and will hold potential in various applications. The approach of combining high-resolution electron microscopy characterizations with molecular dynamics simulations presented above would benefit the understanding of helium ion-matter interactions and contribute to the development of related applications.

## 4. Conclusions

This paper investigates the milling of (0001) single-crystal GaN using the FIB technique with different ion sources (Ga-FIB, Xe-FIB, He-FIB) utilizing experiments and molecular dynamics simulations. The trenches with smaller opening widths and higher aspect ratios for the Ga-FIB and the trenches with larger opening widths and lower aspect ratios for the Xe-FIB were observed for the experimental conditions of 30 kV and 42 pA, which is ascribed to the different probe sizes of the ion beams. The MD simulations further indicate that under the same voltage (2 kV), the Xe-FIB might lead to trenches with higher aspect ratios compared to the Ga-FIB while keeping the probe size the same (1 nm). In addition, both the experiments and simulations suggest that under the selected experimental conditions (30 kV and 22 pA), lattice disorder with interstitials is created for He-FIB. The disordered region enlarges with processing time until the top surface ruptures due to the interstitials-induced stress. In summary, molecular dynamics simulations, together with high-resolution TEM characterizations, are synergistically combined to investigate the ion-source-dependent milling processes, which will provide insight into the selection of different FIB techniques to fabricate nanoscale surface structures.

## Data Availability

Not applicable.

## Supplementary Materials

The following supporting information can be downloaded at https://www.mdpi.com/article/10.3390/nano13212898/s1, Figure S1: Secondary electron images of a cleaved silicon edge used as “knife-edge” for profiling of (a) gallium and (b) xenon-focused ion beams (30 kV, 42 pA). Corresponding measurements of pixel value changes across the knife-edge for (c) gallium and (d) xenon FIB; Figure S2: ADF-STEM images and corresponding EDS mappings showing Ga distribution of GaN by (a) Ga-FIB and (b) Xe-FIB; Figure S3: TEM images (a,b) of milling profile by Xe-FIB (30 kV, 42 pA, 10 μm line scan for 10 s) and the corresponding FFTs of different regions in (b) show three regions: Crystalline, Crystalline-Amorphous Transition, Amorphous.
