# Supplementary material for: A Comparative Study of Gallium-, Xenon-, and Helium-Focused Ion Beams for the Milling of GaN
Source: Nanomaterials (Basel). 2023 Nov 3;13(21):2898. doi: 10.3390/nano13212898 (PMC10647709; doi:10.3390/nano13212898)
Supplement: Supplementary file 1 [file nanomaterials-13-02898-s001.zip › nanomaterials-2612603-supplementary.pdf]

Supplementary data

A Comparative Study of Gallium-, Xenon-, and Helium-Focused Ion Beams for the Milling of GaN

Shuai Jiang and Volkan Ortalan *

Department of Materials Science and Engineering, University of Connecticut, Storrs, CT 06226, USA; shuai.2.jiang@uconn.edu

**vortalan@uconn.edu*

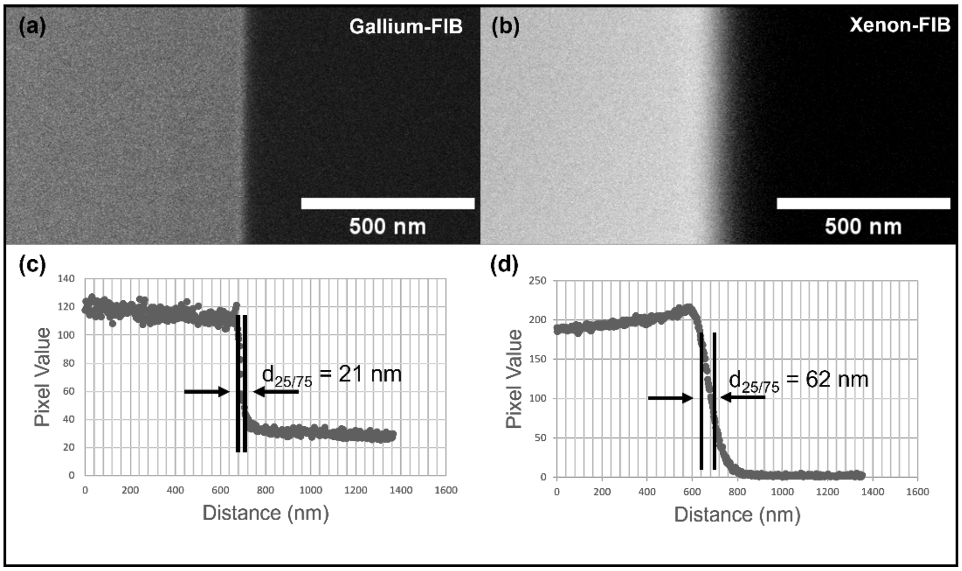

Figure S1. Secondary electron images of a cleaved silicon edge used as “knife-edge” for profiling of (a) gallium and (b) xenon focused ion beams (30 kV, 42 pA). Corresponding measurements of pixel value changes across the knife edge for (c) gallium and (d) xenon FIB.

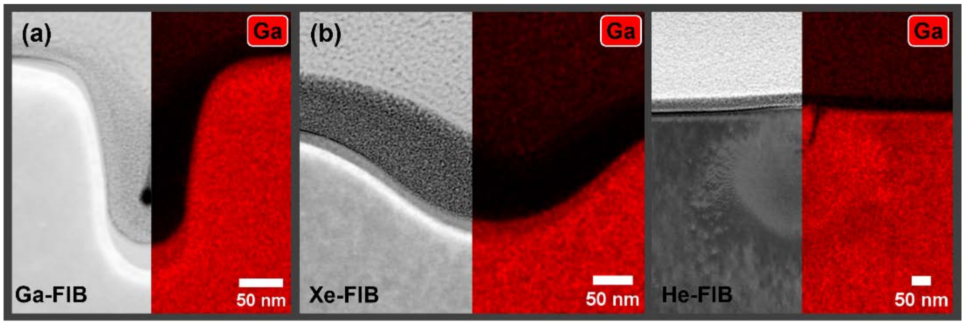

Figure S2. ADF-STEM images and corresponding EDS mappings showing Ga distribution of GaN by (a) Ga-FIB, (b) Xe-FIB and (c) He-FIB.

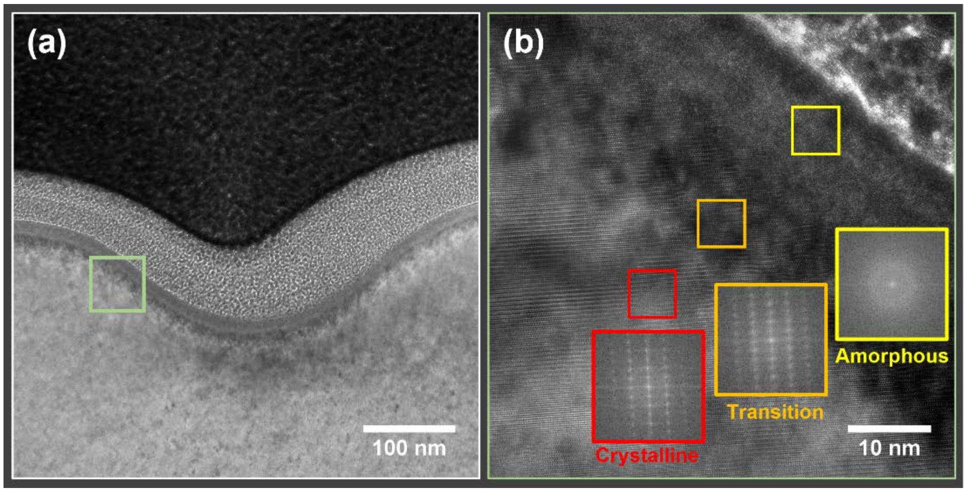

Figure S3. TEM images (a, b) of milling profile by Xe-FIB (30 kV, 42 pA, 10 um line-scan for 10 s) and the corresponding FFTs of different regions in (b) show three regions: Crystalline, Crystalline-Amorphous Transition, Amorphous.
